# The F-box E3 ligase protein FBXO11 regulates EBNA3C-associated degradation of BCL6

**DOI:** 10.1128/jvi.00548-24

**Published:** 2024-06-12

**Authors:** Kunfeng Sun, Dipayan Bose, Rajnish Kumar Singh, Yonggang Pei, Erle S. Robertson

**Affiliations:** 1 The Tumor Virology Program, Department of Otorhinolaryngology-Head and Neck Surgery, Perelman School of Medicine at the University of Pennsylvania, Philadelphia, Pennsylvania, USA; 2 School of Public Health and Emergency Management, Southern University of Science and Technology, Shenzhen, Guangdong, China; 3 Department of Microbiology, Perelman School of Medicine at the University of Pennsylvania, Philadelphia, Pennsylvania, USA; University of Toronto, Toronto, Canada

**Keywords:** Epstein-Barr virus, EBNA3C, FBXO11, E3 ligase, ubiquitin

## Abstract

Most mature B-cell malignancies originate from the malignant transformation of germinal center (GC) B cells. The GC reaction appears to have a role in malignant transformation, in which a major player of the GC reaction is BCL6, a key regulator of this process. We now demonstrate that BCL6 protein levels were dramatically decreased in Epstein-Barr virus (EBV)-positive lymphoblastoid cell lines and Burkitt’s lymphoma cell lines. Notably, BCL6 degradation was significantly enhanced in the presence of both EBNA3C and FBXO11. Furthermore, the amino-terminal domain of EBNA3C, which contains residues 50–100, interacts directly with FBXO11. The expression of EBNA3C and FBXO11 resulted in a significant induction of cell proliferation. Furthermore, BCL6 protein expression levels were regulated by EBNA3C via the Skp Cullin Fbox (SCF)^FBXO11^ complex, which mediated its ubiquitylation, and knockdown of FBXO11 suppressed the transformation of lymphoblastoid cell lines. These data provide new insights into the function of EBNA3C in B-cell transformation during GC reaction and raise the possibility of developing new targeted therapies against EBV-associated cancers.

**IMPORTANCE:**

The novel revelation in our study involves the suppression of BCL6 expression by the essential Epstein-Barr virus (EBV) antigen EBNA3C, shedding new light on our current comprehension of how EBV contributes to lymphomagenesis by impeding the germinal center reaction. It is crucial to note that while several EBV latent proteins are expressed in infected cells, the collaborative mechanisms among these proteins in regulating B-cell development or inducing B-cell lymphoma require additional investigation. Nonetheless, our findings carry significance for the development of emerging strategies aimed at addressing EBV-associated cancers.

## INTRODUCTION

Epstein-Barr virus (EBV), the first human oncogenic virus to be isolated, infects over 95% of adults worldwide and establishes persistent infection throughout life (1, 2). EBV-associated malignancies contribute to approximately 2% of all cancer-related deaths (3). During latent EBV infection, the expression of EBV genes depends on the specific program of latency, which dictates the expression pattern required to drive cell proliferation. Previous studies indicate that during latency III, which is observed in lymphoblastoid cell lines (LCLs), all six Epstein-Barr nuclear antigens (EBNAs 1, 2, 3A, 3B, 3C, and EBNA-LP) as well as latent membrane proteins LMP1 and LMP2 (LMP2A and LMP2B) are expressed (4). This also expressed the EBV small RNAs, which include the EBERs and miRNAs seen in most latency programs (5).

The B-cell lymphoma 6 (BCL6) protein is an important transcriptional repressor that plays a vital role in B-cell development and is involved in regulating immune responses. It is primarily expressed in germinal center (GC) B cells, a specialized region within the secondary lymphoid organs, where B cells undergo intense clonal expansion, somatic hypermutation, and affinity maturation in response to antigens (6). BCL6 prevents premature differentiation of B cells by recruiting co-repressor complexes and chromatin-modifying enzymes to target gene promoters, leading to the suppression of transcription. Importantly, genetic alterations or dysregulation of BCL6 is associated with the development of B-cell lymphomas.

Previously, we reported that EBNA3C regulates BCL6 through two distinct mechanisms. First, it interacts with BCL6, leading to its degradation via the ubiquitin-proteasome pathway (7). Additionally, EBNA3C hinders the transcriptional activity of the BCL6 promoter, resulting in the suppression of BCL6 mRNA expression (8). BCL6 is the product of a proto-oncogene implicated in the pathogenesis of human B-cell lymphomas (9). Recent research demonstrated that BCL6 is a Skp Cullin Fbox E3 ligase substrate and is targeted for ubiquitination and degradation by the SCF complex (9, 10). SCF complexes contain four essential components: Skp1, Cullin, Rbx1/Roc1/Hrt1, and an F-box protein for its E3 ligase activities (11). SCF complexes facilitate interaction between substrates and ubiquitin-conjugating enzymes, which covalently transfer ubiquitin to substrates, which are subsequently degraded. The F-box protein is the subunit of the SCF complex that binds specific substrates, linking it to the complex by binding Skp1 through the F-box (12). The F-box proteins are essential in regulating SCF activity during the cell cycle. The affinity of the F-box protein for protein substrates is regulated through Cdk/cyclin-mediated phosphorylation of target proteins. In normal germinal center B cells, the expression of BCL6 is reduced. However, this signaling pathway is obstructed in a specific subgroup of diffuse large B-cell lymphomas (DLBCLs) due to genetic changes or modifications in the BCL6 gene (13). A subset of DLBCLs demonstrate chromosomal translocations or mutations that disrupt the IRF-responsive region in the BCL6 promoter and block its downregulation by CD40 regulation (14).

F-box proteins are characterized by an amino-terminal 40-residue F-box motif that binds Skp1, followed by protein-protein interaction modules such as leucine-rich repeats or WD (Trp-Asp) repeats that bind substrates (15). There are three classes of F-box proteins: FBXL, FBXW, and FBXO (15). FBXL are proteins containing an F-box and leucine-rich repeats, FBXW are proteins containing an F-box and WD (Trp-Asp) repeats, and FBXO are proteins containing an F-box and either another motif or no other motif (15). FBXO11 is an F-box protein that belongs to the FBXO class, and its dysregulation is linked to oncogenesis (15).

In DLBCL, BCL6 overexpression is observed because of the deletion of FBXO11 (9). When reconstituted, ubiquitination and degradation were promoted and BCL6 levels decreased (9). In EBV-positive DLBCL, BCL6 expression is observed to be frequently decreased (16). Therefore, it was important to investigate whether FBXO11 is responsible for its downregulation due to the pathological differences between EBV-positive and negative DLBCLs. The objective of our present study was to elucidate the regulatory mechanisms involving FBXO11, BCL6, and EBNA3C in contributing to EBV-mediated oncogenesis.

## RESULTS

### The expression of BCL6 is dramatically decreased in the presence of EBNA3C and FBXO11

**Fig 1:**
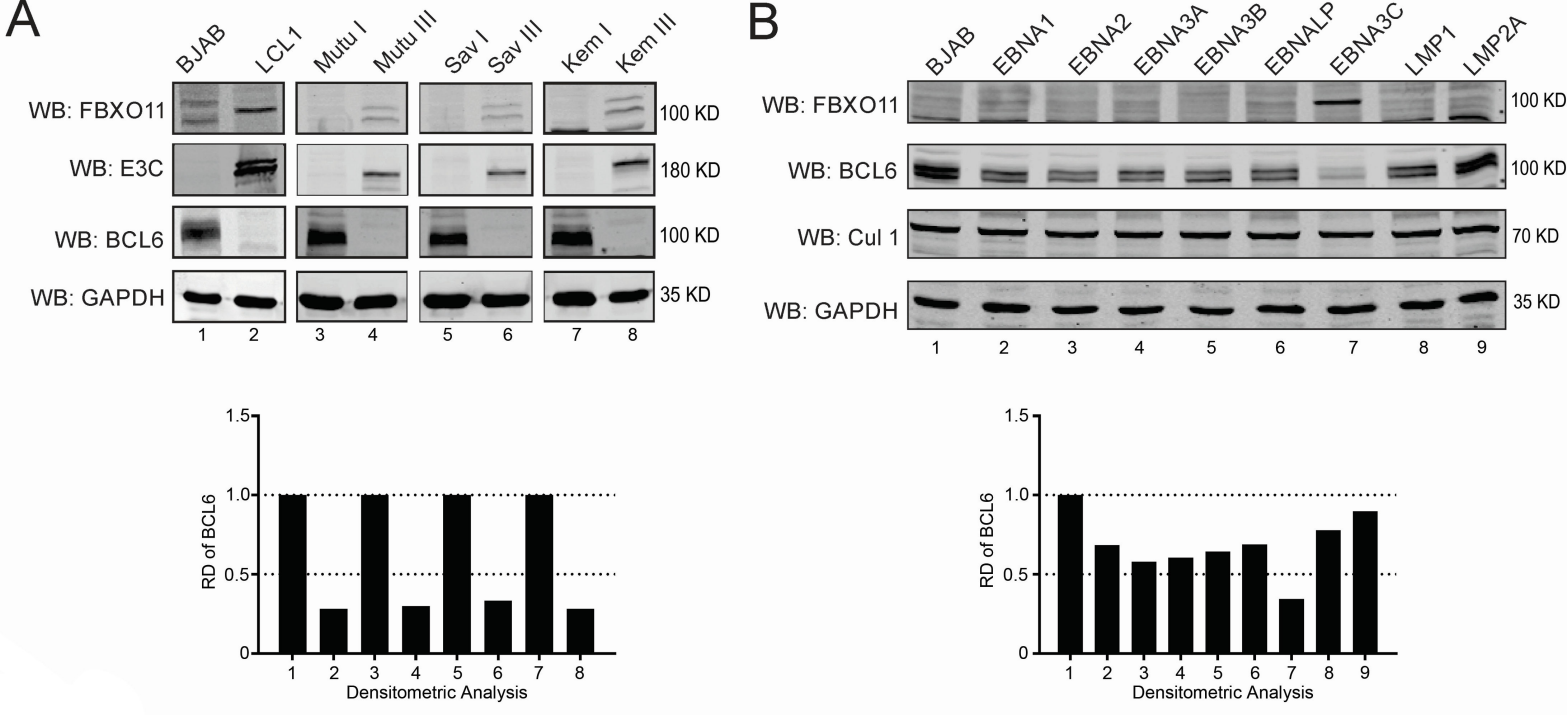
BCL6 level is reduced in latency III EBV-positive cells. (**A**) Fifteen million BJAB, LCL1, Mutu I, Sav I, and Kem I cells and corresponding latency III EBV-positive cells and (**B**) eight latent genes (EBNAs 1, 2, 3A, 3B, 3C, EBNA-LP, LMP1, and LMP2A) expressing stable BJAB cells were harvested and lysed with RIPA buffer. The expression levels of E3C, FBXO11, and BCL6 were detected by western blot. The relative density (RD) of BCL6 was quantitated for a representive blot and shown.

Previously, we reported that during primary EBV infection, the levels of BCL6 were downregulated (8). We also observed that mutant EBV that were devoid of EBNA3C were expressing significantly more BCL6 as compared to the cells infected with wild-type EBV (8). In order to further validate our previous findings and establish a connection between the latency program of EBV and BCL6 expression, we conducted western blot assays to assess the protein levels of BCL6 in various cell lines, both EBV negative and EBV positive. We now show that BCL6 protein levels were dramatically decreased in EBV-positive lymphoblastoid cell lines and Burkitt’s lymphoma cell lines Mutu III, Sav III, and Kem III, which exhibit the latency III program when compared to the same isogenic cells that express the latency I program namely Mutu I, Sav I, and Kem I and the EBV-negative BJAB (Fig. 1A). Densitometric analysis confirmed that the demonstrated drop in protein levels of BCL6 was greater than fivefold as seen in the EBV-positive latency III cells Mutu III, Sav III, and Kem III as compared to that of the EBV-positive latency I cells or EBV-negative cell lines (Fig. 1A). These results suggested that EBV infection was responsible for the reduced expression of BCL6 in human Burkitt’s and lymphoblastoid cell lines. Additionally, the type I latency cells Mutu I, Sav I, and Kem I did not show a reduction in BCL6, strongly suggesting that this may be due to one or more of the latent antigens expressed during the latency III program. To determine which latent gene contributes to the reduction in BCL6 expression levels, western blot analyses were performed with eight latent genes (EBNAs 1, 2, 3A, 3B, 3C, EBNA-LP, LMP1, and LMP2A) stably expressed in BJAB cell lines. The results of the western blot clearly showed that EBNA3C was able to significantly reduce the expression levels of BCL6 by greater than 60% as evidenced by western blot signals and the induced expression of FBXO11 (Fig. 1B, see lane 7 and bottom graph).

**Fig 2:**
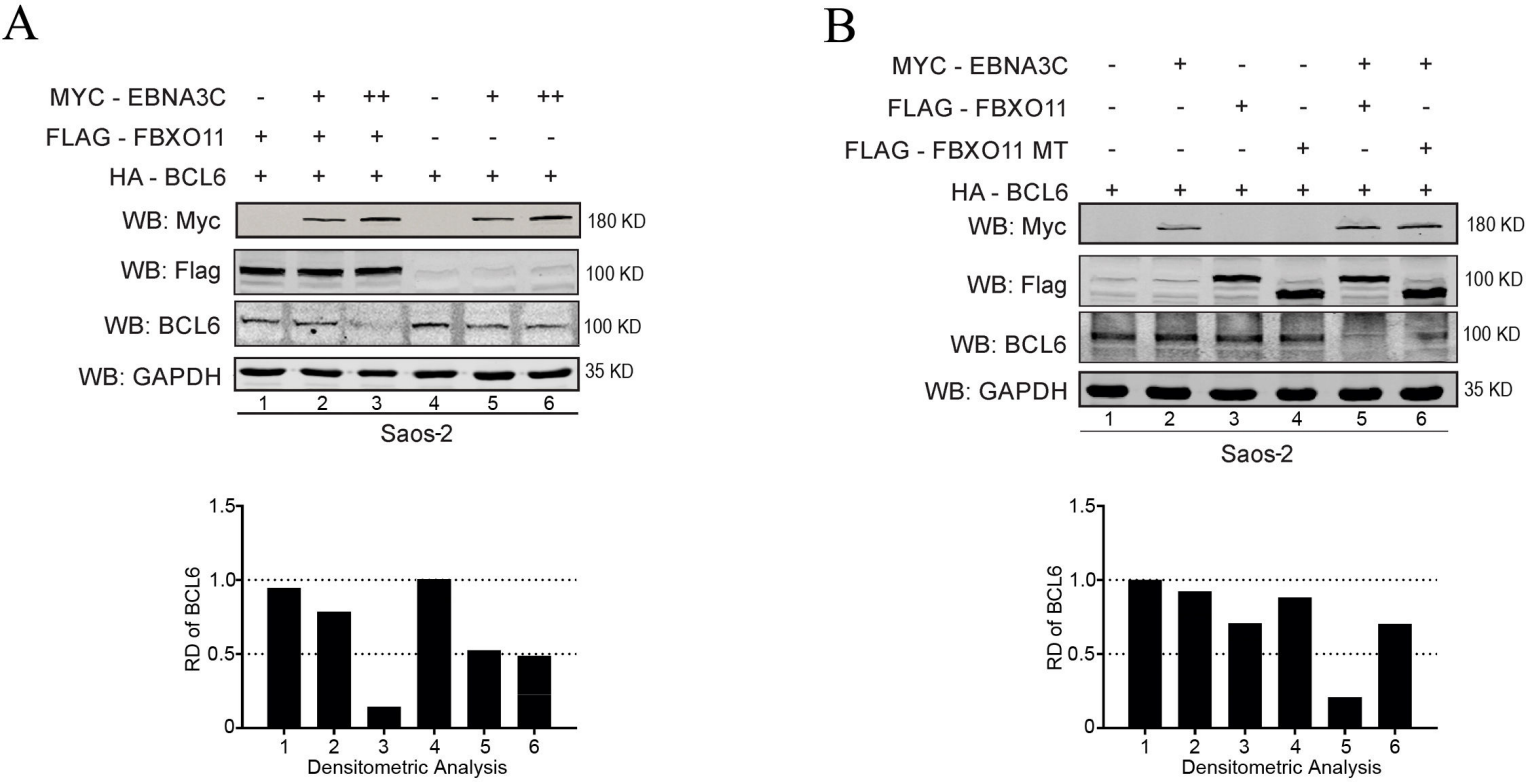
BCL6 levels are decreased in the presence of EBNA3C. Half a million Saos-2 cells were transfected with HA-BCL6 and (**A**) an increasing amount of Myc-tagged EBNA3C (10 and 15 µg) alone or together with Flag-FBXO11, (**B**) Myc-tagged EBNA3C (15 µg), Flag-FBXO11, and Flag-FBXO11mut separately or together. Total amounts of plasmids were kept constant by co-transfecting with the vector. At 48 hours post-transfection, the cells were harvested and lysed with RIPA buffer. The expression levels of E3C, FBXO11, and BCL6 were detected by western blot. The relative density (RD) of BCL6 was quantitated for a representative blot and shown.

To further validate this finding, we exogenously expressed EBNA3C and FBXO11 in Saos-2 cells. We observed that enhanced expression of FBXO11 from a heterologous system in the presence of increasing concentrations of EBNA3C led to a significant downregulation of BCL6 expression levels (Fig. 2A). To further validate these findings in BJAB cells and EBNA3C expressing BJAB7 cells, the cells were transfected with FLAG-FBXO11 and HA-BCL6. We observed significantly lower levels of BCL6 in cells that were expressing EBNA3C and FBXO11 as compared to cells that were expressing either FBXO11 or EBNA3C alone (Fig. S1A). We additionally knocked down the expression of FBXO11 in Saos-2 cells expressing EBNA3C using shRNA and noticed a significant reduction of BCL6 expression (Fig. S1B), which further confirms the involvement of FBXO11 in the regulation of BCL6 protein levels.

To explore the role of FBXO11, we constructed a carboxy-terminal-deleted Flag-tagged FBXO11 mutant and co-transfected it with EBNA3C. The Flag-FBXO11MT is devoid of the carboxy-terminal substrate recognition domain (Δ1,753–2,238 bp). The expression of exogenous EBNA3C was confirmed by western blot using antibody against the c-Myc epitope, while the expression of FBXO11 and FBXO11MT was confirmed by using antibodies against the Flag epitope. We observed that the expression of full-length FBXO11 and EBNA3C led to a dramatic reduction in levels of BCL6 expression, while the exogenous expression of mutant FBXO11 did not show any reduction in levels of BCL6 as a result of its degradation (Fig. 2B, bottom graph). This underlines the importance of FBXO11 and EBNA3C in regulating BCL6 expression levels.

### The amino-terminal domain of EBNA3C binds FBXO11 in human cells

**Fig 3:**
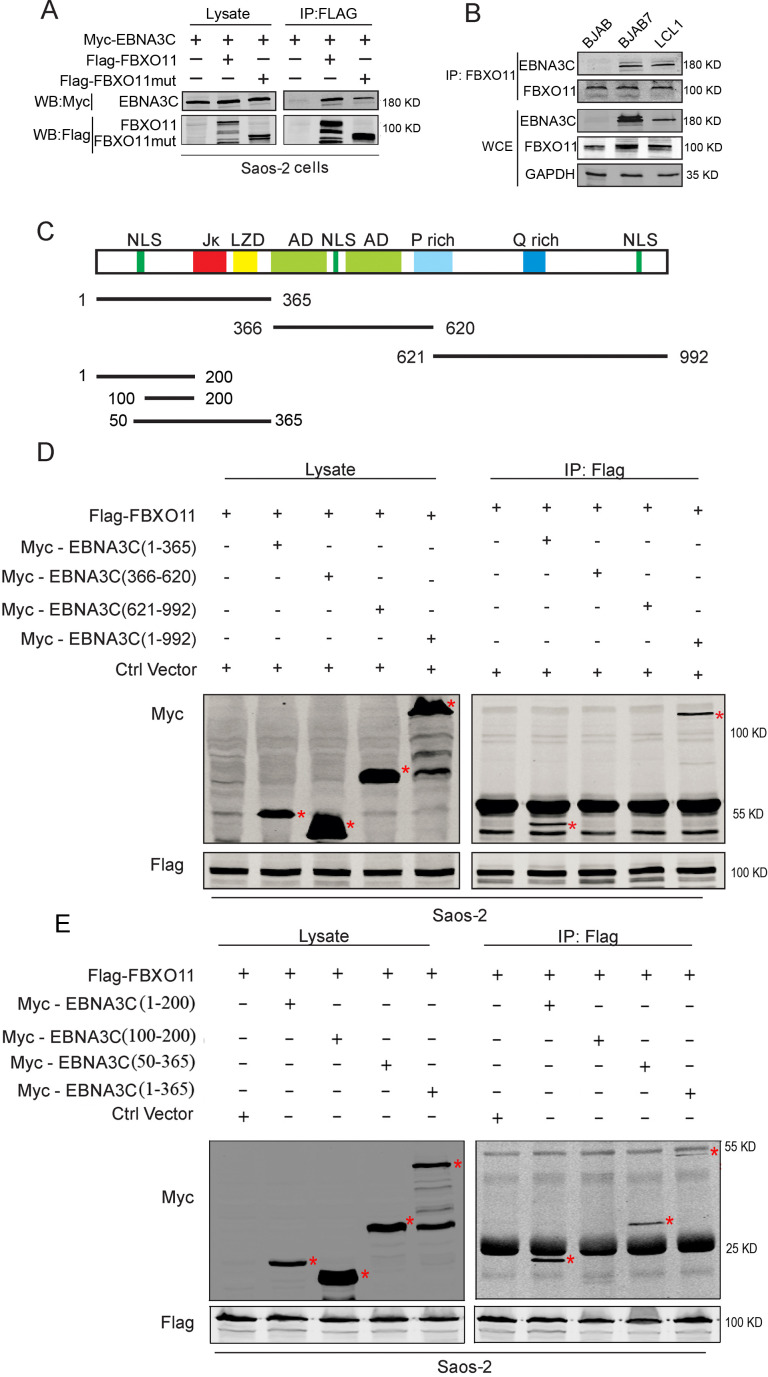
EBNA3C associates with FBXO11 in molecular complexes in human cell lines. (**A**) Ten million Saos-2 cells were transfected with Flag-tagged FBXO11 and Flag-tagged FBXO11mut alone or together with Myc-tagged EBNA3C. At 48 hours post-transfection, the cells were harvested and lysed for immunoprecipitation with 2 µg anti-Flag antibody. The input and immunoprecipitated samples fractionated, and specific signals were detected by western blot by using antibodies against Flag and Myc. (**B**) EBNA3C associated with endogenous FBXO11. Sixty million BJAB, BJAB7, and LCL1 were collected and lysed for immunoprecipitation with 2 µg anti-FBXO11 antibody. Western blot was used to detect specific signals in the inputs and immunoprecipitated samples. (**C**) The schematic diagram summarizes the binding domains between different regions of EBNA3C. Jκ, RBP-Jκ; LZ, leucine zipper domain; AD, acidic domains; P rich, proline-rich; Q rich, glutamine-proline-rich; and NLS, nuclear localization signal. (**D and E**) The N terminus of EBNA3C is critical for EBNA3C and FBXO11 interaction. Ten million Saos-2 cells were transfected with Flag-tagged FBXO11 alone or together with Myc-tagged full-length EBNA3C or EBNA3C truncated mutants. At 48 hours post-transfection, the cells were harvested and lysed for immunoprecipitation with 2 µg anti-Flag antibody. The input and immunoprecipitated samples were resolved using 8% polyacrylamide (D) and 12% polyacrylamide (E) and blotted using specific antibodies.

Next, we examined whether EBNA3C could interact directly with FBXO11. Saos-2 cells were transfected with epitope-tagged Myc-EBNA3C and Flag-FBXO11. Immunoprecipitation with anti-Flag antibody followed by western blot using Myc-specific antibody revealed that Myc-tagged EBNA3C was co-immunoprecipitated with Flag-FBXO11 (Fig. 3A). To further determine the interaction in B-cell lines, BJAB cells, BJAB7 cells that express EBNA3C, and EBV-transformed cells (LCL1) were tested for the interaction of EBNA3C with FBXO11. Western blot analysis demonstrated that endogenous EBNA3C can physically associate with FBXO11 in the background of B cells and more importantly in EBV-transformed lymphoblastoid cell lines (Fig. 3B).

To determine the functional binding domain of EBNA3C that specifically interacts with FBXO11, Myc-tagged full-length and truncated regions of EBNA3C (1–365 aa, 366–620 aa, and 621–992 aa) (Fig. 3C) were co-transfected with Flag-tagged FBXO11 into Saos-2 cells. The targeted protein was immunoprecipitated with anti-Flag-specific antibody. The results demonstrated that FBXO11 was associated with EBNA3C (1–365 aa) along with the full-length EBNA3C protein (1–992 aa) (Fig. 3D). No detectable co-immunoprecipitation was observed with the control vector supporting the associated specificity of the complex between EBNA3C and FBXO11 in these cells. These results showed that EBNA3C amino acid residues 1–365 aa, which include the acidic domain, were responsible for the interaction of EBNA3C with the FBXO11 protein. We further used EBNA3C smaller truncations (1–200 aa, 100–200 aa, and 50–365 aa) to more precisely determine the region of EBNA3C that interacts with FBXO11. We observed Flag-tagged FBXO11 co-immunoprecipitated with 1–200 aa and 50–365 aa fragments of EBNA3C (Fig. 3E). This result clearly demonstrated that the region containing residues 50–100 aa of EBNA3C was critical for binding with FBXO11.

### EBNA3C co-localizes to nuclear compartments with FBXO11 in human cells

**Fig 4:**
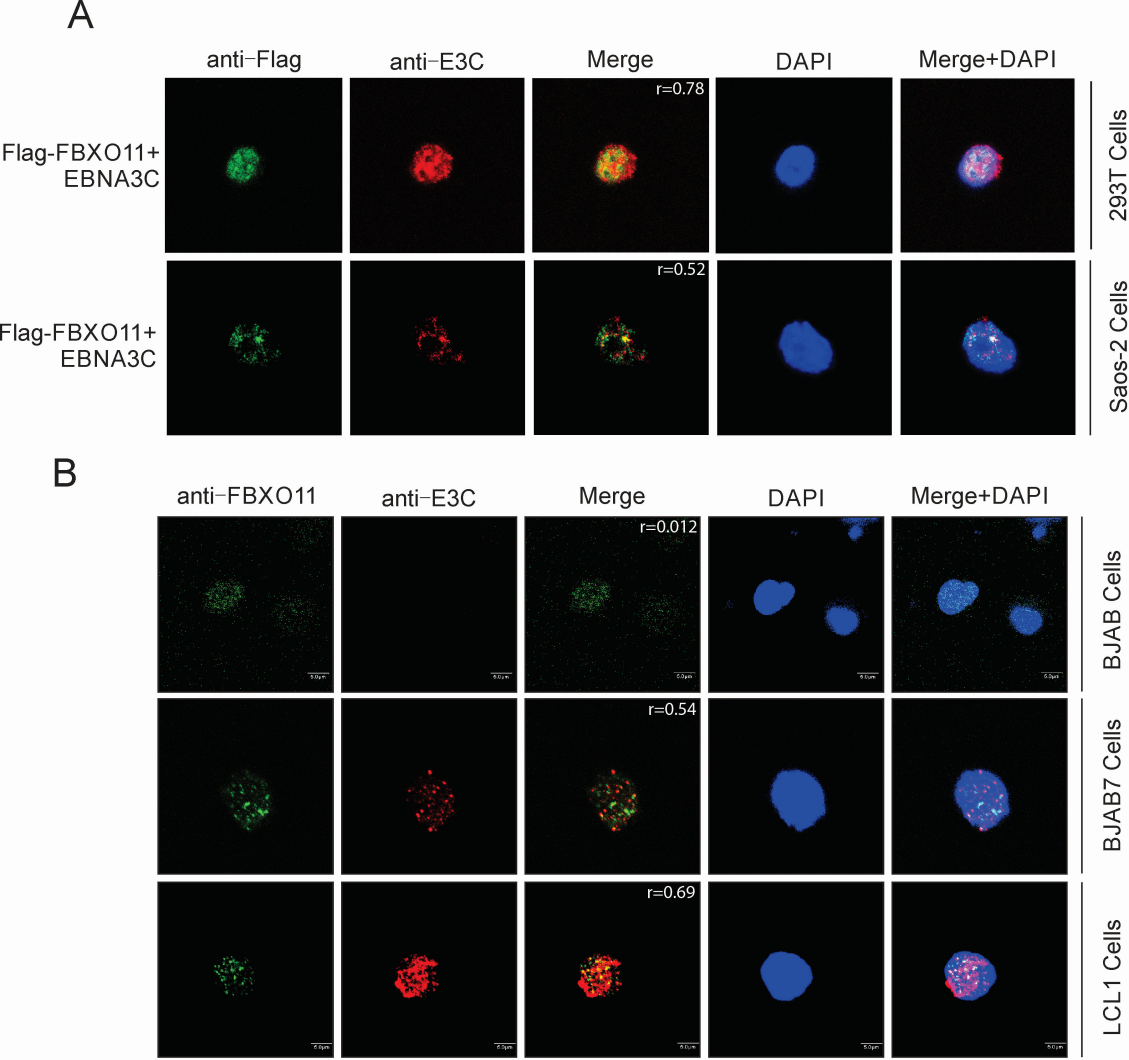
Co-localization of EBNA3C and FBXO11 in human cells. (**A**) EBNA3C co-localized with FBXO11 in 293T and Saos-2 cells. A total of 0.1 million Saos-2 cells were plated on coverslips and transfected with Myc-tagged EBNA3C and Flag-tagged FBXO11. At 24 hours post-transfection, cells were subjected to immunofluorescence assays. (**B**) EBNA3C co-localized with endogenous FBXO11 in B cells. BJAB, BJAB7, and LCL1 cells were plated on the slide and air-dried. The cells were fixed and subjected to immunofluorescence assays as described in Materials and Methods. The colocalization between the green and red channels was calculated using Pearson’s coefficient (*r*) in ImageJ software (17).

To determine the localization of EBNA3C and FBXO11, Saos-2 cells were transfected with constructs expressing Myc-tagged EBNA3C and Flag-tagged FBXO11, and the cellular localization of the expressed proteins was examined using immunofluorescence assays and visualized by fluorescence microscopy. In cells transfected independently with Myc-EBNA3C or Flag-FBXO11 alone, both were found to be primarily expressed in the nucleus (Fig. 4A). In cells co-transfected with Myc-EBNA3C and Flag-FBXO11, the merged yellow fluorescence demonstrated that EBNA3C co-localized with FBXO11 in nuclear compartments in cells expressing these constructs (Fig. 4A).

To further determine the localization of EBNA3C and FBXO11 proteins in more physiologically relevant B cells, immunofluorescence assays were performed using antibodies specific to EBNA3C and FBXO11 proteins. The results further confirmed that EBNA3C co-localized with FBXO11 in nuclear compartments of EBV-transformed cells (Fig. 4B). This was consistent with the results of the above studies, which demonstrated that EBNA3C co-immunoprecipitated with FBXO11 and showed the association of these complexes in the same cellular compartments.

### EBNA3C promotes FBXO11-mediated cell proliferation

**Fig 5:**
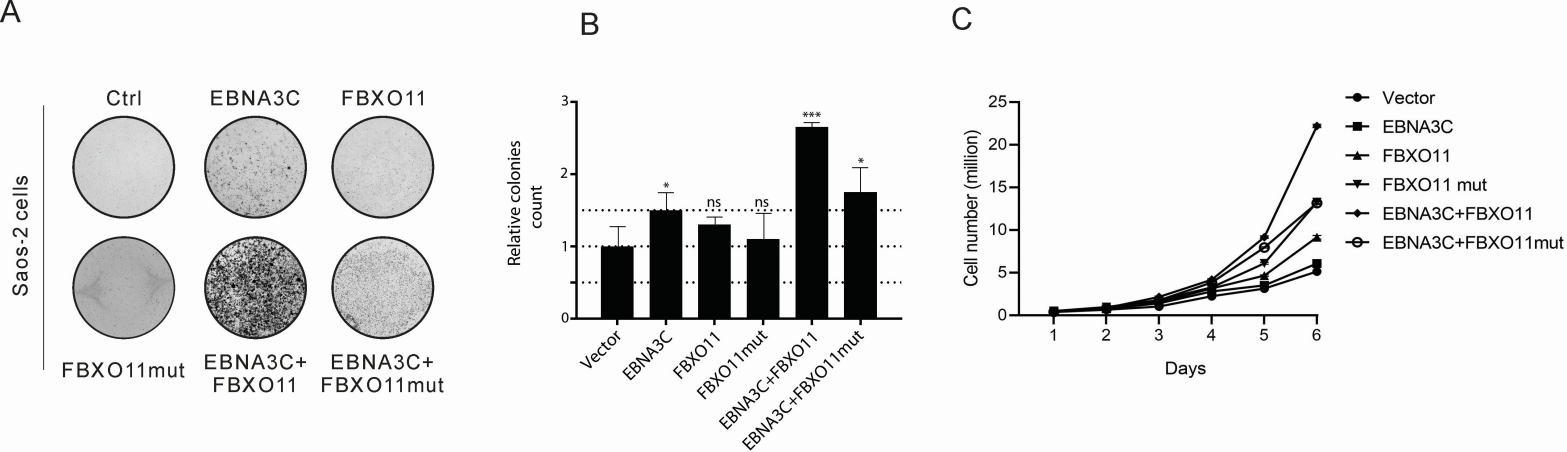
EBNA3C and FBXO11 promote cell proliferation. (**A and B**) Saos-2 cells were transfected with the indicated plasmids and eGFP. The cells were selected with G418 (neomycin) antibiotics for 2 weeks. The cells were fixed, and the cell colonies were stained with 0.1% crystal violet. The relative colony number was measured by Image J software. (**C**) 5 × 10^5^ selected cells were plated and cultured for 6 days. Viable cells were counted every day using trypan blue staining.

Previously, we reported that EBNA3C-mediated regulation of BCL6 promoted cell proliferation by targeting BCL2 and CCND1 (8). Although BCL6 is reported to be an oncoprotein, BCL6 also binds and represses BCL2 and BCL-XL expression (18). To examine the involvement of FBXO11, Saos-2 cells were transfected with expression constructs of EBNA3C and FBXO11 and selected with G418 for 2 weeks to monitor colony formation. We observed a significant increase in colony numbers when EBNA3C and FBXO11 were co-transfected in comparison to those transfected with only EBNA3C or FBXO11 (Fig. 5A and B). We further extended these studies by performing cell proliferation assays as determined by cell counting for 6 days in Saos-2 cells (Fig. 5C). These results demonstrated that expression of EBNA3C and FBXO11 results in a strong induction in cell proliferation as determined by the increase in cell numbers when compared to either EBNA3C or FBXO11 alone and suggests that the complex is important for driving an increase in cell proliferation.

### BCL6 expression is regulated by EBNA3C via SCF^FBXO11^ complex-mediated ubiquitylation

**Fig 6:**
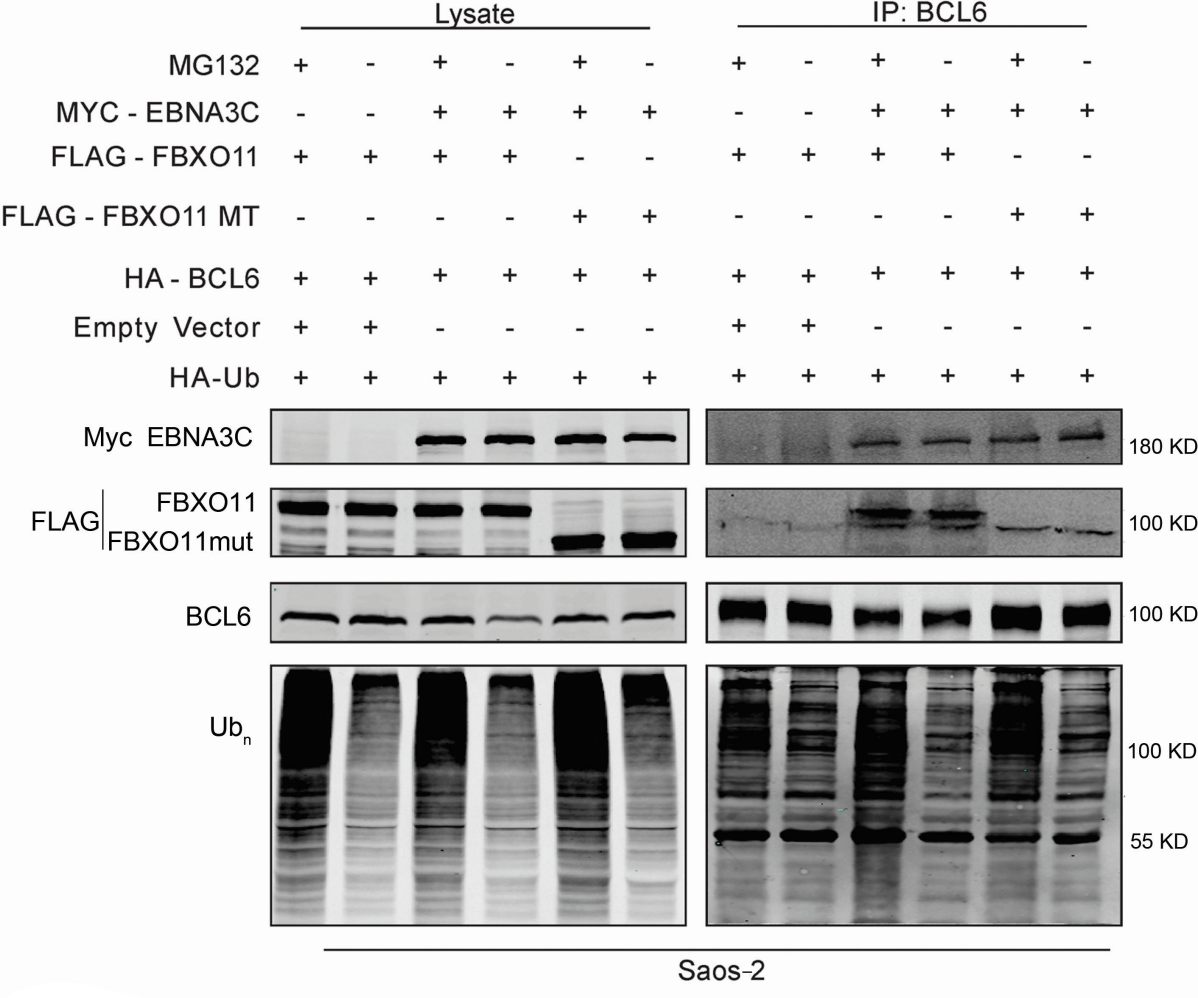
EBNA3C mediates BCL6 degradation through the ubiquitin pathway. EBNA3C enhanced poly-ubiquitination of BCL2. Ten million Saos-2 cells were transfected with the indicated constructs. At 24 hours post-transfection, cells were incubated with 10 µM MG132 for another 16 hours. Then, the cells were harvested and subjected to immunoprecipitation using an antibody against BCL6. The input and immunoprecipitated samples were detected by western blot.

Previous data demonstrated that EBNA3C recruited E3 ligases for targeted degradation of cellular substrates (19, 20). Our previous research has shown that EBNA3C promoted the degradation of the BCL6 protein (8). FBXO11 is a member of the F-box protein family, which is the substrate-binding subunit of the Skp1-Cul1-F-box ubiquitin ligase complex (12). To further assess whether this F-box protein is the E3 Ligase interacting with EBNA3C and BCL6, ubiquitination assays were performed with different expression plasmids for Myc-E3C, Flag-FBXO11, Flag-FBXO11mut, HA-Ub, and HA-BCL6 and incubated for 24 hours followed by MG132 treatment for another 12 hours. This was followed by immunoprecipitation and western blot analysis. The results of this assay demonstrated enhanced ubiquitination of BCL6 when both EBNA3C and FBXO11 were co-expressed as compared to FBXO11 alone or co-expression of FBXO11mut and EBNA3C (Fig. 6). This strongly indicated that BCL6 is likely degraded in the presence of EBNA3C through SCF^FBXO11^ complex-mediated ubiquitylation.

### Knockdown of FBXO11 suppresses the transformation of LCLs

**Fig 7:**
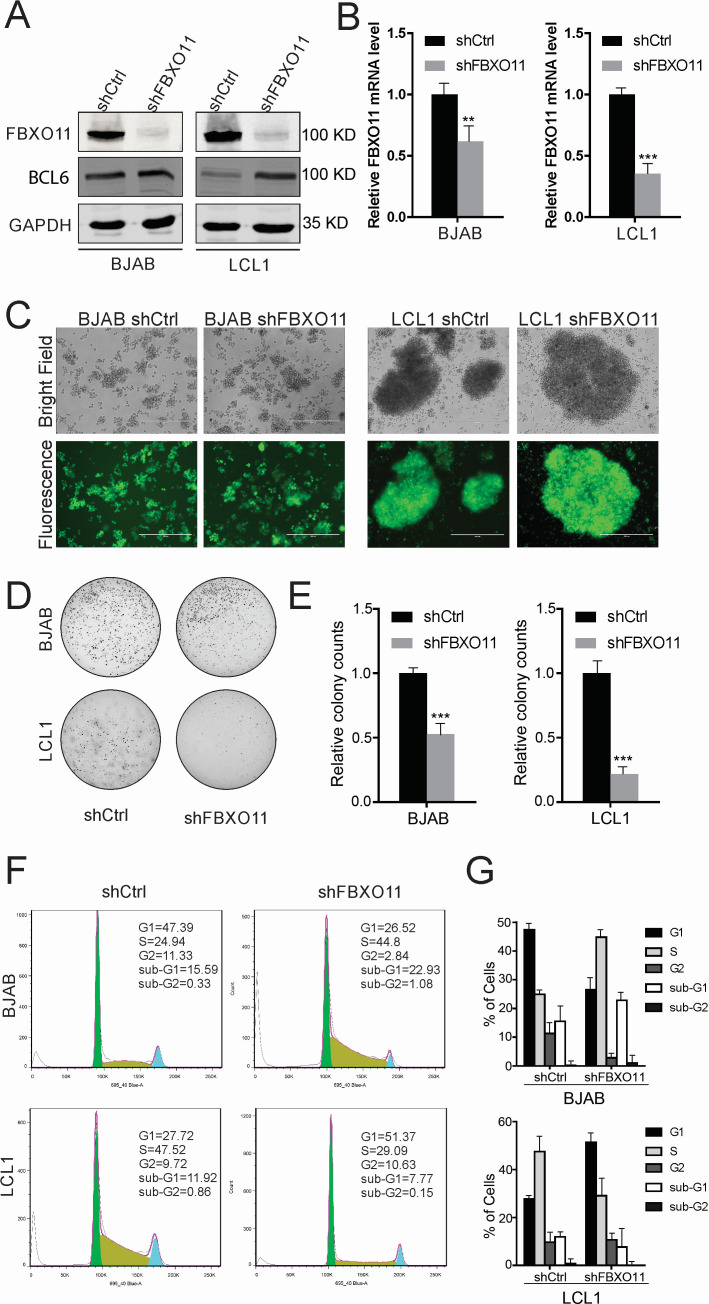
Knockdown of FBXO11 suppresses the transformation activity of LCL1. (**A**)Saos-2 and HEK293 cells were transfected with Flag-tagged FBXO11 and sh-Ctrl, or sh-FBXO11. After 24 hours, the cells were harvested, and the expression of FBXO11 was detected by western blot. (**B**) FBXO11 knockdown (shFBXO11) or control (shCtrl) stable BJAB or LCL1 cells were harvested, and FBXO11 mRNA expression was detected using real-time PCR. (**C**) FBXO11 knocked down BJAB and LCL1 cells were constructed by lentivirus transduction and selected by puromycin for 3 weeks. GFP fluorescence was determined in the selected cells. (**D and E**) Colony formation was measured in FBXO11 knocked down BJAB and LCL1 cells by soft agar assays. The relative colony number was measured by Image J software. (**F and G**) FBXO11 knocked down BJAB and LCL1 cells were stained with PI staining buffer and analyzed by flow cytometry.

To evaluate the effect of FBXO11 on EBNA3C-mediated transformation of B cells, FBXO11 was stably knocked down in BJAB and LCL1 cells by lentivirus transduction using shRNA targeting FBXO11 and selected using puromycin for 3 weeks (Fig. 7A through C). The ability of these selected cell lines to form colonies was determined by soft agar assays. The results showed that a smaller number of colonies were observed in FBXO11 knocked down BJAB cells as compared to the shControl cells (Fig. 7). However, in FBXO11 knocked down EBV-transformed LCL1 cells, the relative colony number was dramatically less (Fig. 7D and E), when compared to the shControl cells. This result clearly demonstrated the significance of EBNA3C and FBXO11 on cell growth and proliferation of EBV-transformed B cells.

To dissect the role of FBXO11 and EBNA3C on the cell cycle, propidium iodide assays were performed on FBXO11 knocked down BJAB and LCL1 cells, and the percentages of cells in different phases of the cell cycle were determined (Fig. 7F and G). The results showed that the knockdown of FBXO11 in BJAB cells increased the percentage of cells in the S phase. However, when FBXO11 was knocked down in LCL1 cells, the percentage of cells in the S phase dramatically decreased. These results indicated that in EBV-infected LCL cells, knockdown of FBXO11 suppressed G1 to S transition, while in EBV-negative BJAB cells, the cell cycle progressed as expected with time.

## DISCUSSION

BCL6 belongs to the BTB/POZ/Zinc Finger protein family and is a nuclear phosphoprotein. It functions as a transcription repressor, suppressing target genes by binding to specific DNA sequences and recruiting corepressors such as SMRT, MTA3, NCoR, and HDAC (21–26). BCL6 plays a crucial role in germinal center formation and somatic hypermutation during B-cell development. Disruptions in the regulatory region of BCL6 caused by chromosomal translocations and mutations result in dysregulation of BCL6 expression, observed in approximately 40% of DLBCL and 5%–10% of FL cases (27). While BCL6 expression is associated with the EBV latent antigens EBNA2 and LMP1, conflicting results have not provided a comprehensive explanation or detailed mechanism for EBV-mediated BCL6 degradation in B-cell lymphoma (28–30). A recent study suggested that EBNA3C has no effect on BCL6 expression (31). However, a previous report indicated that BCL6 expression can increase more than 10-fold in EBNA3C-deleted EBV-infected cells (31, 32). Our previous study conclusively demonstrated that EBNA3C specifically downregulates BCL6 expression at both the transcriptional and post-transcriptional levels (8).

FBXO11 is categorized as a member of the F-box protein family, known for its characteristic F-box domain, which consists of approximately 40 amino acids. This protein family serves as the substrate-binding component within the SCF ubiquitin ligase complex (33). FBXO11 exerts crucial regulatory functions in cell cycle regulation, tumorigenesis, and metastasis of tumor cells through its ability to bind to substrate proteins and facilitate their degradation (34). The SCF^FBXO11^ complex mediates ubiquitylation and degradation of BCL6, a transcription repressor that is required for normal germinal center development (9). FBXO11 interacts with CDT2 (a DCAF protein that controls cell-cycle progression) and recruits CDT2 to the SCF^FBXO11^ complex to promote its proteasomal degradation (35, 36). FBXO11 was shown to mediate the ubiquitylation and degradation of SNAIL, a transcription factor that promotes epithelial-mesenchymal transition (EMT) (37). The recognition of SNAIL by FBXO11 appears to be dependent on Ser-11 phosphorylation of SNAIL by protein kinase D1 (PDK1). FBXO11 blocks SNAIL-induced EMT, tumor initiation, and metastasis in multiple breast cancer models (37).

**Fig 8:**
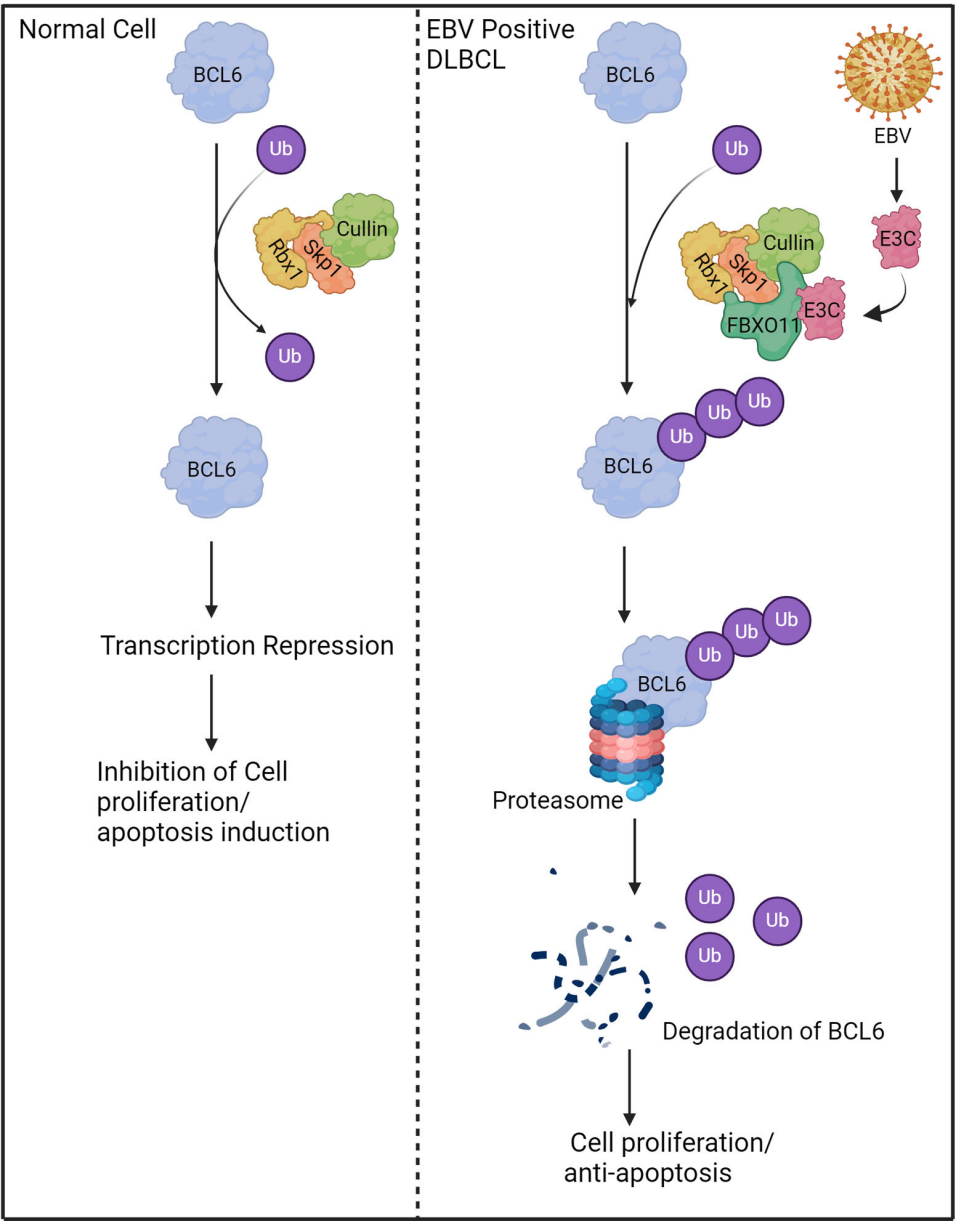
Schematic diagram illustrating how EBNA3C promotes the degradation of FBXO11. In normal cells, the levels of FBXO11 were maintained to a minimal level that led to the restoration of BCL6 levels within the cell. In DLBCL, FBXO11 interacts with EBNA3C and acts as E3 ubiquitin ligase to polyubiquitinylate and degrades BCL6.

In the present study, we demonstrated that EBNA3C specifically interacts with FBXO11 and mediates the degradation of BCL6 through the ubiquitin-proteasome dependent pathway (Fig. 8). A previous study showed that the BCL6 protein can be targeted for degradation by cellular factor FBXO11 in DLBCL (9). In our study, we have successfully demonstrated that the recruitment of FBXO11 and degradation of BCL6 by EBNA3C require this E3 ligase. Earlier research suggests that acetylation of BCL6 within its PEST domain leads to the inactivation of its co-repressor recruitment function (38). Moreover, the activation of the MAPK signaling pathway induces the phosphorylation of BCL6, subsequently resulting in its degradation through the ubiquitin-proteasome pathway (39). Additional investigations are needed to ascertain the potential connections between EBNA3C-mediated BCL6 degradation, BCL6 acetylation, and phosphorylation.

The significance of BCL6’s role in GC B cells is evident from its ability to regulate multiple functional pathways within the cell. BCL6 has been found to target over 1,000 genes by binding to their promoters, thereby influencing downstream signaling pathways crucial for GC development. These pathways are involved in various cellular processes such as apoptosis, cell cycle regulation, and cell differentiation (40, 41). One of the important proteins targeted by BCL6 is BCL2, which plays a critical role in anti-apoptosis. BCL6 directly interacts with Miz1 and binds to the BCL2 promoter, resulting in the inhibition of Miz1-induced transcriptional activity of BCL2 in B cells (42). Dysregulation of BCL6-mediated BCL2 expression is a common occurrence in DLBCL and FL (43). Our previous findings demonstrated that EBNA3C induces cell proliferation by the degradation of BCL6, thus inhibiting its expression, thereby alleviating the suppression of BCL2 and activating the anti-apoptosis pathway for tumorigenesis (8). Additionally, CCND1, a direct BCL6 target in human B cells, is de-repressed, promoting G1-S transition in EBV-transformed LCLs. The control of other cyclin proteins by BCL6 remains unclear. Notably, studies suggest that CCND2 is another BCL6 target, but its expression is negatively correlated (44–48). EBNA3C upregulates the expression of activation-induced cytidine deaminase (AID), responsible for somatic hypermutation and class-switch recombination, in EBV-infected cells (31, 49). BCL6 can also enhance AID expression by inhibiting miR-155 and miR-361 (50). The regulation of AID expression by EBNA3C without BCL6 involvement warrants further exploration (51). Recent research identified BCL6-targeted genes in T follicular helper cells, underscoring BCL6’s genome-wide occupancy and role in transcriptional regulatory networks (52). Although BCL6 small-molecular inhibitors show promise as therapeutic targets for human lymphomas (53), the BCL6-mediated regulatory networks in EBV-transformed LCLs remain elusive. Xenografts of LCLs in BCL6 knockout mice may elucidate BCL6’s biological function in EBV-related lymphomagenesis. However, a more efficient *in vivo* model is crucial for uncovering the roles of EBNA3C and other latent antigens in the GC reaction.

In summary, the inhibition of BCL6 expression by the essential EBV antigen EBNA3C provides a novel perspective on EBV’s role in lymphomagenesis by impeding the GC reaction. While various EBV latent proteins are expressed in infected cells, understanding how these proteins collaborate to regulate B-cell development or induce B-cell lymphoma requires further investigation. Nevertheless, our findings have implications for emerging strategies targeting EBV-associated cancers.

## MATERIALS AND METHODS

### Cell culture and plasmid constructs

EBV-negative Burkitt’s lymphoma BJAB cells were kindly provided by Elliot Kieff (Harvard Medical School, Boston, MA, USA). Mutu I and Mutu III cells were kindly provided by Yan Yuan (School of Dental Medicine, University of Pennsylvania, Philadelphia, PA, USA). Sav I and Sav III cell lines, Kem I and Kem III cell lines were kindly provided by Dr. Paul M. Lieberman (The Wistar Institute, Philadelphia, PA, USA). BJAB stably expressing EBNA3C cells (BJAB7 and BJAB10) were prepared by transfecting pZipneo EBNA3C into BJAB cells, followed by neomycin selection (54). LCL1 cells were EBV-transformed immortalized LCLs generated in our laboratory (55).

Cells were grown at 37°C with 5% CO_2_ in RPMI medium containing 7% bovine growth serum (BGS), penicillin (100 units/mL), and streptomycin (0.1 mg/mL). BJAB-KSHV cells were maintained with additional selection using puromycin (2 µg/mL). Human Embryonic Kidney cell line (HEK 293T) was obtained from Jon Aster (Brigham and Women’s Hospital, Boston, MA, USA), and Saos-2 cells were grown in DMEM medium containing 10% BGS with antibiotics at the above concentration.

Plasmids expressing full-length EBNA3C or its truncations such as 1–365, 366–620, and 621–992 with C-terminal Myc-tag have been previously described (56). HA-tagged full-length BCL6 were kindly provided by Dr. Riccardo Dalla-Favera (Columbia University, New York, USA). Plasmid expressing the full-length FBXO11 was cloned by using Kpn1 and BamH1 restriction enzyme in the pA3F vector as previously described (56).

### Immunoblotting

Cells were lysed with RIPA buffer (10 mM Tris, pH 7.5, 1% Nonidet-P40, 2 mM EDTA, and 150 mM NaCl plus protease inhibitors), and protein concentration was determined using Bradford assays. The lysates were analyzed by Western blots using the appropriate primary antibodies and conjugated fluorescent secondary antibodies. The results were scanned with an Odyssey Infrared scanner. Densitometry analysis was performed with the Odyssey V3.0 software. The western blots were repeated three times, and the quantification is a representation of one of the assays based on densitometry.

### Confocal microscopy

Saos-2 cells were cultivated in 12-well plates on coverslips and then transfected with the specified plasmids. After 24 hours of transfection, the cells were washed three times with ice-cold PBS and fixed using 4% paraformaldehyde. B cells, totaling 2 million, were harvested, washed with PBS, and air-dried. These cells were also fixed with 4% paraformaldehyde at room temperature for 20 minutes. Subsequently, the fixed cells were washed with PBS and permeabilized with 0.2% Triton X-100 for 20 minutes at room temperature. To block nonspecific binding, the cells were treated with 3% bovine serum albumin for 0.5 hour at room temperature. Next, the cells were incubated overnight at 4°C with either rabbit anti-EBNA3C antibody, mouse anti-FBXO11 antibody, or mouse anti-Flag antibody. Following this incubation, the cells were washed three times with PBS and then incubated with secondary antibodies: Alexa Fluor 594 goat anti-rabbit (ThermoFisher; 1:250) or Alexa Fluor 488 goat anti-mouse (ThermoFisher 1:250) for 1 hour at room temperature. To visualize the nuclei, the cells were stained with DAPI (4′,6-diamidino-2-phenylindole) for 10 minutes. After another three washes with PBS, the coverslips were flipped over and placed on a glass slide with a drop of mounting media. Confocal images were captured using a Fluoview FV300 confocal microscope. The images were edited in ImageJ, and the co-localization was calculated using Pearson’s coefficient (*r*) and was presented in the figure. “*r*” value ranges from 0 to 1, where 0 represents no co-localization, while 1 represents perfect co-localization (17).

### Colony formation assay

Saos-2 cells and HEK293 in 6-well plates were transfected with GFP, control vector, Flag-FBXO11, Flag-FBXO11mut alone, or together with Myc-EBNA3C. The transfected cells were selected in DMEM with 2 mg/mL G418 (Sigma-Aldrich, St. Louis, MO, USA). Two weeks later, 4% paraformaldehyde was used to fix the cell colonies at room temperature for 30 minutes and stained with 0.1% crystal violet for 0.5 hour. The colonies were scanned by BioRAD ChemiDOC MP Imaging system, and the relative colony number was measured by Image J software. All assays were repeated three times for reproducibility.

### Generation of lentiviral particles

ShControl and shFBXO11 lentiviruses were produced through the transfection of HEK293T cells with transfer plasmids, along with third-generation packaging and envelop plasmids, following previously described protocols (57, 58). Briefly, HEK293T cells were cultured in 10 cm cell culture dishes until reaching 40%–60% confluency. Transfection involved the use of 10 µg of transfer plasmids along with packaging and envelop plasmids, employing the calcium phosphate method. After discarding the initial culture medium containing the transfection mix, the supernatants were collected at 12-hour intervals between 24 and 96 hours. These supernatants were then filtered through a 0.45 µm syringe filter, and lentiviruses were concentrated via ultracentrifugation at 23,500 rpm for 2 hours (59). The resulting pelleted lentiviruses were resuspended in 1 mL of complete medium and stored frozen until needed for transduction. Transduction was carried out by mixing cells with the resuspended lentiviral stock in the presence of 8 µg/mL polybrene. Cells were subjected to selection with 2 µg/mL puromycin 48 hours post-transduction.

### Soft agar assays

The soft agar assays were performed in FBXO11 knocked down BJAB or LCL1 cells. One milliliter of 0.5% agar supplemented with RPMI media was poured into a 6-well plate and set aside to solidify. 1 × 10^5^ cells were mixed with 0.5 mL of 0.3% agar/medium and poured on top of the 0.5% agar layer. Two weeks later, colonies were stained with 0.005% crystal violet overnight and scanned using the BioRAD ChemiDOC MP Imaging system. The relative colony numbers were measured by Image J software. All assays were repeated three times for reproducibility.

### Flow cytometry

Flow cytometry was performed with FBXO11 knocked down BJAB or LCL1 cells to detect the cell cycle and DNA content. One million stable cells were collected and suspended with 300 µL of PBS containing 2% FBS. Then, the cells were fixed with 1 mL ice-cold 100% ethanol for 24 hours at 4°C. After washing with PBS containing 2% FBS once, the cells were stained with PI staining buffer (0.5 mg/mL propidium iodide in PBS and 50 µg/mL RNase A) for 30 minutes at room temperature and analyzed using a FACS Calibur (BD LSR II Special Order System, USA) and FlowJo software (Treestar, Inc., San Carlos, CA, USA).

### Statistical analysis

Each experiment was repeated at least three times. The mean scores were examined by using Student’s *t*-test. All statistical tests were performed using Microsoft Office Excel. A *P*-value of 0.05 was considered to be a statistically significant difference. A *P*-value of 0.01 indicated high statistical significance.

## Data Availability

The authors confirm that the data supporting the findings of this study are available on request if required.

## SUPPLEMENTAL MATERIAL

Fig. S1 jvi.00548-24-s0001.docx Effect of EBNA3C on BCL6 expression in BJAB cells, and expression of FBXO11 and BCL6 in Saos-2 cells.

ASM does not own the copyrights to Supplemental Material that may be linked to, or accessed through, an article. The authors have granted ASM a non-exclusive, world-wide license to publish the Supplemental Material files. Please contact the corresponding author directly for reuse.

## References

[1] de-The G, Day NE, Geser A, Lavoue MF, Ho JH, Simons MJ, Sohier R, Tukei P, Vonka V, Zavadova H. 1975. Sero-epidemiology of the Epstein-Barr virus: preliminary analysis of an international study - a review. IARC Sci Publ:3–16. 191375

[2] Babcock GJ, Decker LL, Volk M, Thorley-Lawson DA. 1998. EBV persistence in memory B cells in vivo. Immunity 9:395–404. doi:10.1016/s1074-7613(00)80622-6 9768759

[3] Khan G, Hashim MJ. 2014. Global burden of deaths from Epstein-Barr virus attributable malignancies 1990-2010. Infect Agent Cancer 9:38. doi:10.1186/1750-9378-9-38 25473414 PMC4253616

[4] Young LS, Yap LF, Murray PG. 2016. Epstein-Barr virus: more than 50 years old and still providing surprises. Nat Rev Cancer 16:789–802. doi:10.1038/nrc.2016.92 27687982

[5] Kang MS, Kieff E. 2015. Epstein-Barr virus latent genes. Exp Mol Med 47:e131. doi:10.1038/emm.2014.84 25613728 PMC4314583

[6] Mlynarczyk C, Fontán L, Melnick A. 2019. Germinal center-derived lymphomas: the darkest side of humoral immunity. Immunol Rev 288:214–239. doi:10.1111/imr.12755 30874354 PMC6518944

[7] Pei Y, Robertson ES. 2022. The central role of the ubiquitin-proteasome system in EBV-mediated oncogenesis. Cancers (Basel) 14:611. doi:10.3390/cancers14030611 35158879 PMC8833352

[8] Pei Y, Banerjee S, Jha HC, Sun Z, Robertson ES. 2017. An essential EBV latent antigen 3C binds Bcl6 for targeted degradation and cell proliferation. PLoS Pathog 13:e1006500. doi:10.1371/journal.ppat.1006500 28738086 PMC5524291

[9] Duan S, Cermak L, Pagan JK, Rossi M, Martinengo C, di Celle PF, Chapuy B, Shipp M, Chiarle R, Pagano M. 2012. FBXO11 targets BCL6 for degradation and is inactivated in diffuse large B-cell lymphomas. Nature 481:90–93. doi:10.1038/nature10688 22113614 PMC3344385

[10] Pighi C, Cheong T-C, Compagno M, Patrucco E, Arigoni M, Olivero M, Wang Q, López C, Bernhart SH, Grande BM, et al.. 2021. Frequent mutations of FBXO11 highlight BCL6 as a therapeutic target in Burkitt lymphoma. Blood Adv 5:5239–5257. doi:10.1182/bloodadvances.2021005682 34625792 PMC9153037

[11] Xie CM, Wei W, Sun Y. 2013. Role of SKP1-CUL1-F-box-protein (SCF) E3 ubiquitin ligases in skin cancer. J Genet Genomics 40:97–106. doi:10.1016/j.jgg.2013.02.001 23522382 PMC3861240

[12] Skaar JR, Pagan JK, Pagano M. 2013. Mechanisms and function of substrate recruitment by F-box proteins. Nat Rev Mol Cell Biol 14:369–381. doi:10.1038/nrm3582 23657496 PMC3827686

[13] Winter JN, Weller EA, Horning SJ, Krajewska M, Variakojis D, Habermann TM, Fisher RI, Kurtin PJ, Macon WR, Chhanabhai M, Felgar RE, Hsi ED, Medeiros LJ, Weick JK, Reed JC, Gascoyne RD. 2006. Prognostic significance of Bcl-6 protein expression in DLBCL treated with CHOP or R-CHOP: a prospective correlative study. Blood 107:4207–4213. doi:10.1182/blood-2005-10-4222 16449523 PMC1895783

[14] Saito M, Gao J, Basso K, Kitagawa Y, Smith PM, Bhagat G, Pernis A, Pasqualucci L, Dalla-Favera R. 2007. A signaling pathway mediating downregulation of BCL6 in germinal center B cells is blocked by BCL6 gene alterations in B cell lymphoma. Cancer Cell 12:280–292. doi:10.1016/j.ccr.2007.08.011 17785208

[15] Kipreos ET, Pagano M. 2000. The F-box protein family. Genome Biol 1:REVIEWS3002. doi:10.1186/gb-2000-1-5-reviews3002 11178263 PMC138887

[16] Ok CY, Li L, Xu-Monette ZY, Visco C, Tzankov A, Manyam GC, Montes-Moreno S, Dybkaer K, Chiu A, Orazi A, et al.. 2014. Prevalence and clinical implications of Epstein-Barr virus infection in de novo diffuse large B-cell lymphoma in Western countries. Clin Cancer Res 20:2338–2349. doi:10.1158/1078-0432.CCR-13-3157 24583797 PMC4014309

[17] Dunn KW, Kamocka MM, McDonald JH. 2011. A practical guide to evaluating colocalization in biological microscopy. Am J Physiol Cell Physiol 300:C723–C742. doi:10.1152/ajpcell.00462.2010 21209361 PMC3074624

[18] Cardenas MG, Oswald E, Yu W, Xue F, MacKerell AD, Melnick AM. 2017. The expanding role of the BCL6 oncoprotein as a cancer therapeutic target. Clin Cancer Res 23:885–893. doi:10.1158/1078-0432.CCR-16-2071 27881582 PMC5315622

[19] Knight JS, Sharma N, Robertson ES. 2005. Epstein-Barr virus latent antigen 3C can mediate the degradation of the retinoblastoma protein through an SCF cellular ubiquitin ligase. Proc Natl Acad Sci U S A 102:18562–18566. doi:10.1073/pnas.0503886102 16352731 PMC1317900

[20] Saha A, Murakami M, Kumar P, Bajaj B, Sims K, Robertson ES. 2009. Epstein-Barr virus nuclear antigen 3C augments Mdm2-mediated p53 ubiquitination and degradation by deubiquitinating Mdm2. J Virol 83:4652–4669. doi:10.1128/JVI.02408-08 19244339 PMC2668485

[21] Ye BH, Rao PH, Chaganti RS, Dalla-Favera R. 1993. Cloning of bcl-6, the locus involved in chromosome translocations affecting band 3q27 in B-cell lymphoma. Cancer Res 53:2732–2735. 8504412

[22] Ye BH, Lista F, Lo Coco F, Knowles DM, Offit K, Chaganti RS, Dalla-Favera R. 1993. Alterations of a zinc finger-encoding gene, BCL-6, in diffuse large-cell lymphoma. Science 262:747–750. doi:10.1126/science.8235596 8235596

[23] Dhordain P, Lin RJ, Quief S, Lantoine D, Kerckaert JP, Evans RM, Albagli O. 1998. The LAZ3(BCL-6) oncoprotein recruits a SMRT/mSIN3A/histone deacetylase containing complex to mediate transcriptional repression. Nucleic Acids Res 26:4645–4651. doi:10.1093/nar/26.20.4645 9753732 PMC147883

[24] Dhordain P, Albagli O, Lin RJ, Ansieau S, Quief S, Leutz A, Kerckaert JP, Evans RM, Leprince D. 1997. Corepressor SMRT binds the BTB/POZ repressing domain of the LAZ3/BCL6 oncoprotein. Proc Natl Acad Sci U S A 94:10762–10767. doi:10.1073/pnas.94.20.10762 9380707 PMC23478

[25] Fujita N, Jaye DL, Geigerman C, Akyildiz A, Mooney MR, Boss JM, Wade PA. 2004. MTA3 and the Mi-2/NuRD complex regulate cell fate during B lymphocyte differentiation. Cell 119:75–86. doi:10.1016/j.cell.2004.09.014 15454082

[26] Huynh KD, Bardwell VJ. 1998. The BCL-6 POZ domain and other POZ domains interact with the co-repressors N-CoR and SMRT. Oncogene 17:2473–2484. doi:10.1038/sj.onc.1202197 9824158

[27] Basso K, Dalla-Favera R. 2012. Roles of BCL6 in normal and transformed germinal center B cells. Immunol Rev 247:172–183. doi:10.1111/j.1600-065X.2012.01112.x 22500840

[28] Carbone A, Gaidano G, Gloghini A, Pastore C, Saglio G, Tirelli U, Dalla-Favera R, Falini B. 1997. BCL-6 protein expression in AIDS-related non-Hodgkin's lymphomas: inverse relationship with Epstein-Barr virus-encoded latent membrane protein-1 expression. Am J Pathol 150:155–165. 9006332 PMC1858533

[29] Martín-Pérez D, Vargiu P, Montes-Moreno S, León EA, Rodríguez-Pinilla SM, Lisio LD, Martínez N, Rodríguez R, Mollejo M, Castellvi J, Pisano DG, Sánchez-Beato M, Piris MA. 2012. Epstein-Barr virus microRNAs repress BCL6 expression in diffuse large B-cell lymphoma. Leukemia 26:180–183. doi:10.1038/leu.2011.189 21788950

[30] Boccellato F, Anastasiadou E, Rosato P, Kempkes B, Frati L, Faggioni A, Trivedi P. 2007. EBNA2 interferes with the germinal center phenotype by downregulating BCL6 and TCL1 in non-Hodgkin's lymphoma cells. J Virol 81:2274–2282. doi:10.1128/JVI.01822-06 17151114 PMC1865942

[31] Kalchschmidt JS, Bashford-Rogers R, Paschos K, Gillman ACT, Styles CT, Kellam P, Allday MJ. 2016. Epstein-Barr virus nuclear protein EBNA3C directly induces expression of AID and somatic mutations in B cells. J Exp Med 213:921–928. doi:10.1084/jem.20160120 27217538 PMC4886369

[32] White RE, Groves IJ, Turro E, Yee J, Kremmer E, Allday MJ. 2010. Extensive co-operation between the Epstein-Barr virus EBNA3 proteins in the manipulation of host gene expression and epigenetic chromatin modification. PLoS One 5:e13979. doi:10.1371/journal.pone.0013979 21085583 PMC2981562

[33] Zheng N, Schulman BA, Song L, Miller JJ, Jeffrey PD, Wang P, Chu C, Koepp DM, Elledge SJ, Pagano M, Conaway RC, Conaway JW, Harper JW, Pavletich NP. 2002. Structure of the Cul1-Rbx1-Skp1-F boxSkp2 SCF ubiquitin ligase complex. Nature 416:703–709. doi:10.1038/416703a 11961546

[34] Schneider C, Kon N, Amadori L, Shen Q, Schwartz FH, Tischler B, Bossennec M, Dominguez-Sola D, Bhagat G, Gu W, Basso K, Dalla-Favera R. 2016. FBXO11 inactivation leads to abnormal germinal-center formation and lymphoproliferative disease. Blood 128:660–666. doi:10.1182/blood-2015-11-684357 27166359 PMC9709922

[35] Rossi M, Duan S, Jeong YT, Horn M, Saraf A, Florens L, Washburn MP, Antebi A, Pagano M. 2013. Regulation of the CRL4^Cdt2^ ubiquitin ligase and cell-cycle exit by the SCF^Fbxo11^ ubiquitin ligase. Mol Cell 49:1159–1166. doi:10.1016/j.molcel.2013.02.004 23478441 PMC3624904

[36] Abbas T, Mueller AC, Shibata E, Keaton M, Rossi M, Dutta A. 2013. CRL1-FBXO11 promotes Cdt2 ubiquitylation and degradation and regulates Pr-Set7/Set8-mediated cellular migration. Mol Cell 49:1147–1158. doi:10.1016/j.molcel.2013.02.003 23478445 PMC3615078

[37] Zheng H, Shen M, Zha YL, Li W, Wei Y, Blanco MA, Ren G, Zhou T, Storz P, Wang HY, Kang Y. 2014. PKD1 phosphorylation-dependent degradation of SNAIL by SCF-FBXO11 regulates epithelial-mesenchymal transition and metastasis. Cancer Cell 26:358–373. doi:10.1016/j.ccr.2014.07.022 25203322 PMC4159622

[38] Bereshchenko OR, Gu W, Dalla-Favera R. 2002. Acetylation inactivates the transcriptional repressor BCL6. Nat Genet 32:606–613. doi:10.1038/ng1018 12402037

[39] Niu H, Ye BH, Dalla-Favera R. 1998. Antigen receptor signaling induces MAP kinase-mediated phosphorylation and degradation of the BCL-6 transcription factor. Genes Dev 12:1953–1961. doi:10.1101/gad.12.13.1953 9649500 PMC316953

[40] Basso K, Saito M, Sumazin P, Margolin AA, Wang K, Lim WK, Kitagawa Y, Schneider C, Alvarez MJ, Califano A, Dalla-Favera R. 2010. Integrated biochemical and computational approach identifies BCL6 direct target genes controlling multiple pathways in normal germinal center B cells. Blood 115:975–984. doi:10.1182/blood-2009-06-227017 19965633 PMC2817639

[41] Ci W, Polo JM, Cerchietti L, Shaknovich R, Wang L, Yang SN, Ye K, Farinha P, Horsman DE, Gascoyne RD, Elemento O, Melnick A. 2009. The BCL6 transcriptional program features repression of multiple oncogenes in primary B cells and is deregulated in DLBCL. Blood 113:5536–5548. doi:10.1182/blood-2008-12-193037 19307668 PMC2689052

[42] Saito M, Novak U, Piovan E, Basso K, Sumazin P, Schneider C, Crespo M, Shen Q, Bhagat G, Califano A, Chadburn A, Pasqualucci L, Dalla-Favera R. 2009. BCL6 suppression of BCL2 via Miz1 and its disruption in diffuse large B cell lymphoma. Proc Natl Acad Sci U S A 106:11294–11299. doi:10.1073/pnas.0903854106 19549844 PMC2708681

[43] Yang H, Green MR. 2019. Epigenetic programing of B-cell lymphoma by BCL6 and its genetic deregulation. Front Cell Dev Biol 7:272. doi:10.3389/fcell.2019.00272 31788471 PMC6853842

[44] Kinugasa Y, Hieda M, Hori M, Higashiyama S. 2007. The carboxyl-terminal fragment of pro-HB-EGF reverses Bcl6-mediated gene repression. J Biol Chem 282:14797–14806. doi:10.1074/jbc.M611036200 17392284

[45] Nahar R, Ramezani-Rad P, Mossner M, Duy C, Cerchietti L, Geng H, Dovat S, Jumaa H, Ye BH, Melnick A, Müschen M. 2011. Pre-B cell receptor-mediated activation of Bcl6 induces pre-B cell quiescence through transcriptional repression of MYC. Blood 118:4174–4178. doi:10.1182/blood-2011-01-331181 21856866 PMC3204735

[46] Kusam S, Vasanwala FH, Dent AL. 2004. Transcriptional repressor BCL-6 immortalizes germinal center-like B cells in the absence of p53 function. Oncogene 23:839–844. doi:10.1038/sj.onc.1207065 14737119

[47] Wei F, Zaprazna K, Wang J, Atchison ML. 2009. PU.1 can recruit BCL6 to DNA to repress gene expression in germinal center B cells. Mol Cell Biol 29:4612–4622. doi:10.1128/MCB.00234-09 19564417 PMC2725722

[48] Chevallier N, Corcoran CM, Lennon C, Hyjek E, Chadburn A, Bardwell VJ, Licht JD, Melnick A. 2004. ETO protein of t(8;21) AML is a corepressor for Bcl-6 B-cell lymphoma oncoprotein. Blood 103:1454–1463. doi:10.1182/blood-2003-06-2081 14551142

[49] Pasqualucci L, Bhagat G, Jankovic M, Compagno M, Smith P, Muramatsu M, Honjo T, Morse HC, Nussenzweig MC, Dalla-Favera R. 2008. AID is required for germinal center-derived lymphomagenesis. Nat Genet 40:108–112. doi:10.1038/ng.2007.35 18066064

[50] Basso K, Schneider C, Shen Q, Holmes AB, Setty M, Leslie C, Dalla-Favera R. 2012. BCL6 positively regulates AID and germinal center gene expression via repression of miR-155. J Exp Med 209:2455–2465. doi:10.1084/jem.20121387 23166356 PMC3526356

[51] Basso K, Dalla-Favera R. 2015. Germinal centres and B cell lymphomagenesis. Nat Rev Immunol 15:172–184. doi:10.1038/nri3814 25712152

[52] Liu Y, Yang W, Pan Y, Ji J, Lu Z, Ke Y. 2016. Genome-wide analysis of Epstein-Barr virus (EBV) isolated from EBV-associated gastric carcinoma (EBVaGC). Oncotarget 7:4903–4914. doi:10.18632/oncotarget.6751 26716899 PMC4826252

[53] Cardenas MG, Yu W, Beguelin W, Teater MR, Geng H, Goldstein RL, Oswald E, Hatzi K, Yang SN, Cohen J, Shaknovich R, Vanommeslaeghe K, Cheng H, Liang D, Cho HJ, Abbott J, Tam W, Du W, Leonard JP, Elemento O, Cerchietti L, Cierpicki T, Xue F, MacKerell AD, Melnick AM. 2016. Rationally designed BCL6 inhibitors target activated B cell diffuse large B cell lymphoma. J Clin Invest 126:3351–3362. doi:10.1172/JCI85795 27482887 PMC5004937

[54] Robertson ES, Grossman S, Johannsen E, Miller C, Lin J, Tomkinson B, Kieff E. 1995. Epstein-Barr virus nuclear protein 3C modulates transcription through interaction with the sequence-specific DNA-binding protein J kappa. J Virol 69:3108–3116. doi:10.1128/JVI.69.5.3108-3116.1995 7707539 PMC189012

[55] Cotter MA, Robertson ES. 2000. Modulation of histone acetyltransferase activity through interaction of epstein-barr nuclear antigen 3C with prothymosin alpha. Mol Cell Biol 20:5722–5735. doi:10.1128/MCB.20.15.5722-5735.2000 10891508 PMC86050

[56] Banerjee S, Lu J, Cai Q, Saha A, Jha HC, Dzeng RK, Robertson ES. 2013. The EBV latent antigen 3C inhibits apoptosis through targeted regulation of interferon regulatory factors 4 and 8. PLoS Pathog 9:e1003314. doi:10.1371/journal.ppat.1003314 23658517 PMC3642079

[57] Cai Q, Verma SC, Choi JY, Ma M, Robertson ES. 2010. Kaposi's sarcoma-associated herpesvirus inhibits interleukin-4-mediated STAT6 phosphorylation to regulate apoptosis and maintain latency. J Virol 84:11134–11144. doi:10.1128/JVI.01293-10 20719954 PMC2953196

[58] Singh RK, Lang F, Pei Y, Jha HC, Robertson ES. 2018. Metabolic reprogramming of Kaposi's sarcoma associated herpes virus infected B-cells in hypoxia. PLoS Pathog 14:e1007062. doi:10.1371/journal.ppat.1007062 29746587 PMC5963815

[59] Jha HC, Lu J, Verma SC, Banerjee S, Mehta D, Robertson ES. 2014. Kaposi's sarcoma-associated herpesvirus genome programming during the early stages of primary infection of peripheral blood mononuclear cells. mBio 5:e02261-14. doi:10.1128/mBio.02261-14 25516617 PMC4271552

