# Supplementary material for: The F-box E3 ligase protein FBXO11 regulates EBNA3C-associated degradation of BCL6
Source: J Virol. 2024 Jun 12;98(7):e00548-24. doi: 10.1128/jvi.00548-24 (PMC11265398; doi:10.1128/jvi.00548-24)
Supplement: Fig. S1 — Effect of EBNA3C on BCL6 expression in BJAB cells, and expression of FBXO11 and BCL6 in Saos-2 cells. [file jvi.00548-24-s0001.docx]

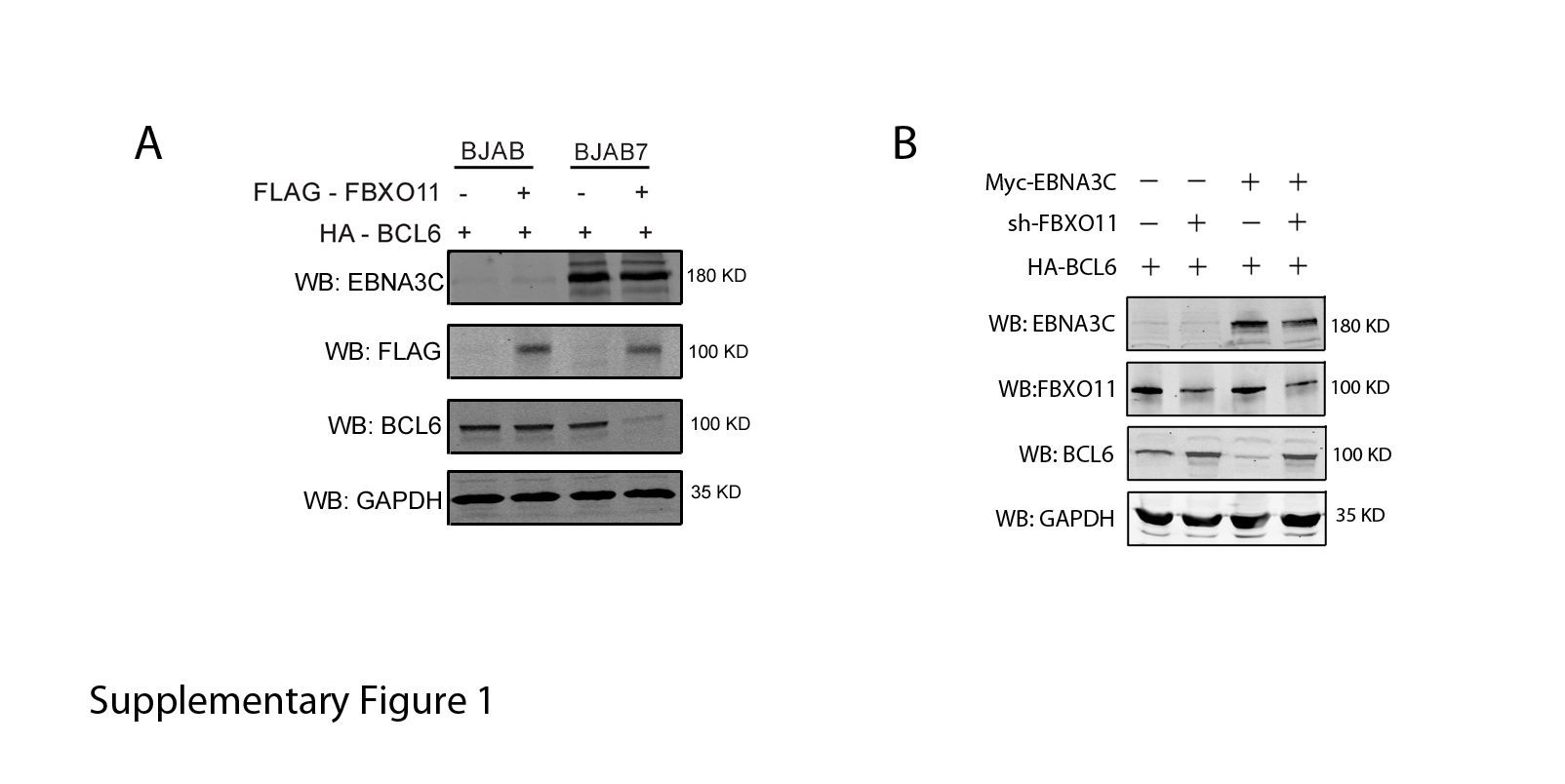
**Supplemental information**

**Supplementary Figure 1:** (A) Effect of EBNA3C on BCL6 expression in BJAB cells. BJAB and BJAB7 cells were transfected with FLAG-FBXO11 and with HA-BCL6. Western blot was performed to determine for the protein levels of BCL6, EBNA3C and FBXO11. GAPDH was used as a loading control.

(B) Saos-2 cells were transfected with EBNA3C, sh-FBXO11 and HA-BCL6 and the expression of FBXO11 and BCL6 was tested. GAPDH was used as loading control.
